# Discharge criteria, practices, and decision-making in the transition of preterm infants to home

**DOI:** 10.1038/s41390-024-03752-w

**Published:** 2024-11-27

**Authors:** Sofia Arwehed, Anna Axelin, Johan Ågren, Ylva Thernström Blomqvist

**Affiliations:** 1 Department of Women’s and Children’s Health, Uppsala University, Uppsala, Sweden; 2 Center for Research and Development, Region Gävleborg/Uppsala University, Gävle, Sweden; 3 Department of Nursing Science, University of Turku, Turku, Finland

## Abstract

**Background:**

Early discharge to neonatal home care is common practice for preterm infants in Sweden but the evidence base for assessing infant and parent readiness is limited and there are no nationally defined discharge guidelines or criteria. To investigate potential facilitators and barriers in the transition to home, we examined discharge criteria, pre- and post-discharge practices, and staff decision-making.

**Methods:**

All (*n* = 36) Swedish units participated in this descriptive mixed method study based on semi-structured interviews with one physician and one registered nurse representing each unit.

**Results:**

Discharge criteria and practices varied, both between and within units. Staff were ambivalent about the timing of discharge and postponed giving discharge-related information to parents. The transition process was staff-driven, with limited parental involvement in care planning, and staff discontinuity delayed discharge. Home care combining telemedicine and home visits, adapted to the needs and preference of the family, was considered effective and appraised. Socially vulnerable families or those with limited language proficiency had restricted access to homecare.

**Conclusions:**

There is a need for improved standardization of, and parental involvement in discharge planning for preterm infants. Earlier transfer of care responsibilities to parents should facilitate transition to home and shorten length of hospital stay.

**Impact:**

- Our findings provide insight into facilitators and barriers in preterm infants’ transition from hospital to home.
- Staff were ambivalent about timing of discharge, and criteria and practices varied between and within units depending on local routines and staff preferences.
- The transition process was staff-driven, with limited parental involvement in care planning, and staff discontinuity caused delay.
- Home care models combining telemedicine and home visits, adapted to the needs of the family, was described as effective and appraised.
- Empowering parents by earlier transfer of care responsibilities and involvement in care planning, could facilitate transition to home and reduce length of stay.

## Introduction

Nearly two million infants are born very preterm, i.e. at less than 32 weeks’ gestation, each year.^1^ They spend weeks to months in the neonatal unit due to immature breathing, thermoregulation, and feeding,^2^ and many parents experience stress due to the perceived vulnerability and prognosis of their infant.^3,4^ Parent–infant separation is common during hospitalization for reasons mainly related to suboptimal unit design and staff-centered routines.^5,6^ The hospital environment including its specialized staff, can be intimidating for parents seeking to develop their parental role, and gain confidence in caring for and emotionally bonding with their infant.^5,7^ Even with optimal support, parents have described their journey through birth, hospitalization, and subsequent home care, as a period of ‘suspended parenthood’.^8,9^ To minimize separation and alleviate stress, the care process should at all times promote parental presence, involvement and sharing of power and responsiblity.^6,10–12^ By earlier acquisition of independence, such a principle can also facilitate the transition home and early discharge. The concept of neonatal home care (NH) includes a range of measures to support families caring for their preterm infant at home, such as telemedicine, staff visiting at home or outpatient visits, and combinations thereof.^13–17^

While early discharge to NH, including with enteral feeding tube, is common practice in most Swedish units, there are currently no nationally defined discharge criteria or guidelines. The evidence base for the assessment of infant and parental discharge readiness is limited, and practice highly variable.^17^ The present investigation aimed to understand potential facilitators and barriers in transitioning to NH by querying pre-discharge preparation, staff discharge decision-making and practice, and post-discharge support in Swedish neonatal care units.

## Methods

### Design and participants

We performed a descriptive mixed method study with convergent parallel design.^18^ Study information and a request to interview a physician and a registered nurse (RN) responsible for discharge and home care routines, was e-mailed to the medical directors of Sweden’s neonatal units (*n* = 36, University hospitals; *n* = 7, County hospitals; *n* = 29). All agreed to participate, study information was repeated to the designated respondents, and individual consent was obtained. All respondents were involved in patient care, responsible for discharge and home care protocols, and made discharge decisions on a regular basis. Early discharge was defined as discharge from the neonatal unit to home while an infant still requires tube feeding and neonatal staff support.

### Data collection and analysis

A semi-structured interview guide (S1) was developed by the authors based on previous literature and experience on discharge and home care practices for preterm infants. Four main areas were covered: (a) discharge criteria and process; (b) pre-discharge preparation and information given to parents; (c) parental involvement in the unit and; (d) support at home. After a pilot test interview, two open-ended questions about facilitators of and barriers to earlier discharge were added. Joint interviews were held in with the physician and RN in all units except one, where only the physician could participate. There was a single combined interview for the three units at Karolinska University Hospital and a single combined for the two units at Skåne University Hospital, resulting in thirty-three interviews representing thirty-six units. All interviews were conducted by the first author (SA) between February and December 2021 and lasted 50–70 min. Each interview began with the collection of quantitative data, and local guidelines were gathered. The interviews were recorded, transcribed, and analyzed by the first author (SA), using qualitative content analysis according to Graneheim, Lindgren, and Lundman.^19,20^ Meaning units were identified and designated code words describing their main content. These codes were subsequently sorted by their similarities and differences related to the aim of the study and then stepwise abstracted into sub-categories, main categories, and theme. Initially, five interviews were coded, analyzed and evaluated with the co-authors (AA and YTB), after which the remaining interviews were coded and analyzed. The analyze and results were re-evaluated among the authors several times, resulting in consensus regarding the final categorization and theme.

## Results

Details of the participating units’ discharge criteria, pre-discharge parent education, and structure of home care support are presented in Tables 1–3. Qualitative content analysis identified one theme related to staff challenges in determining when to transition infant care from hospital to home, and five categories describing decision-making in this process (Table 4).

**Table 1:** Discharge criteria.

	All units *n* = 36	University Hospitals *n* = 7	County Hospitals *n* = 29
Postmenstrual age			
Not specified	5 (14)	0	5 (17)
≥33 weeks	6 (17)	2 (29)	4 (14)
≥34 weeks	18 (50)	5 (71)	13 (45)
≥35 weeks	7 (19)	0	7 (24)
Maintain normal body temperature without heated mattress	36 (100)	7 (100)	29 (100)
Cardiopulmonary stable (free from apnea)			
Caffeine discontinued before discharge as standard practice	36 (100)	7 (100)	29 (100)
Having routines for discontinuing caffeine at home	4 (11)	2 (29)	2 (7)
Home monitoring			
Apnea alarm/Pulse oximetry if PMA < 35 weeks	8 (22)	2 (29)	6 (21)
Body weight			
Not specified	29 (81)	5 (71)	24 (83)
≥1500–1800 g	7 (19)	2 (29)	5 (17)
Feeding skills			
Remain respiratory stable while sucking/swallowing	25 (69)	6 (86)	19 (66)
Breast or bottle feed specified amount	9 (25)	1 (14)	8 (27)
Fully breast or bottle fed (no feeding tube)	2 (6)	0	2 (7)
Parents			
Swedish or English speaking	16 (44)	3 (43)	13 (45)
Access to car	8 (22)	1 (14)	7 (24)
Home <10 km from hospital	4 (11)	1 (14)	3 (10)
Non-smoking	11 (31)	2 (29)	9 (31)
Pre-discharge test			
Car seat test	4 (11)	2 (29)	2 (7)
Structured evaluation of pulse oximetry, respiration and ECG over a set time	8 (22)	2 (29)	6 (21)

All numbers are *n*(%).

**Table 2:** Parental pre-discharge preparation.

	All units *n* = 36	University Hospitals *n* = 7	County Hospitals *n* = 29
Elements of standardized training			
Cardiopulmonary resuscitation	33 (85)	7 (100)	26 (90)
Safe handling of feeding tube (incl. check tube placement)	34 (94)	7 (100)	27 (93)
Reposition feeding tube	28 (78)	7 (100)	21 (72)
Administer oral medications	36 (100)	7 (100)	29 (100)
Safe sleep/SIDS prevention	35 (97)	7 (100)	28 (97)
Assess infant status and signs of deterioration	36 (100)	7 (100)	29 (100)
Perform emergency call (non-native speaking parents)	13 (36)	3 (43)	10 (34)

All numbers are *n*(%).

**Table 3:** Neonatal home care characteristics.

	All units *n* = 34	University Hospitals *n* = 7	County Hospitals *n* = 27
Staffing			
Registered nurses only	14 (41)	3 (43)	11 (41)
Assistant nurses only	3 (9)	0	3 (11)
Both	17 (50)	4 (57)	13 (48)
Additional resources at weekly or bi-weekly rounds			
Pediatrician/Neonatologist	32 (94)	7 (100)	25 (93)
Nutritionist	3 (9)	0	3 (11)
Physiotherapist	1 (3)	0	1 (4)
Medical Social worker	3 (9)	0	3 (11)
No formal rounds	2 (6)	0	2 (7)
Structure of home care			
Outpatient re-visits	34 (100)	7 (100)	27 (100)
Home visits	24 (71)	7 (100)	17 (63)
Telemedicine	14 (41)	4 (57)	10 (37)
Round-the-clock phone support and NICU readmission	34 (100)	7 (100)	27 (100)
Equipment at home			
Scale	26 (76)	7 (100)	19 (70)
Breast pump	34 (100)	7 (100)	27 (100)
Apnea alarm/Pulse oximetry (if PMA < 35 weeks)	8 (24)	2 (29)	6 (22)

All numbers are *n*(%).

**Table 4:** Theme, categories, and subcategories identified in qualitative content analysis.

Theme	Categories	Subcategories
Hospital or home?	Staff ambivalence	Conflicting values and responsibilities
		What type of care can be moved to home?
	Infant assessment	Evaluation of physiological stability
		Feeding and nutrition
	Evaluation of parent’s competence	Vague criteria and guiding documents
		Balancing infant and parent readiness
		Social aspects
	Staff-driven process for the family’s transition to home	Provision of parental information and training
		Skills training
		Flexible but non-uniform discharge planning
	Support at home	Adjust to being at home
		Supportive practices at home

### The challenge of discharge timing

Respondents expressed ambivalence about the optimal timing of discharge in relation to the infant’s physiological stability, parents’ role and readiness, and the appropriate level of care at home. Assessment criteria and tools varied (Table 1), were perceived to be of limited value, and lacking evidence base, leaving the evaluation to be mainly based on bedside assessments and interactions with the family. Individual staff’s experience and preferences greatly influenced the evaluation, which in combination with varying discharge criteria and guidelines, resulted in a variable and non-systematic length of stay. Socially vulnerable families and those with limited language proficiency had restricted access to homecare.

Parental presence and transfer of care responsibilities were facilitated when moving to a family room. While parents were gradually handed responsibilities in daily routine care, the respondents reported limited sharing of power and parental involvement in longer term care planning. Almost uniformly, the discharge planning was staff-initiated and driven, and there were both organizational and family-oriented motives for accelerating or delaying the process. There was a time gap in discharge information and preparation, initially provided at admission and then often postponed until the infant met discharge criteria, which respondents noted left parents with limited time to adjust. It was repeatedly described how parents at the start of discharge preparation could express anxiety but also a strong desire to take their infant home, if given the necessary support and reassurance of safety.

“*Often when parents are faced with the idea of transitioning to homecare, their reaction is ‘oh no, that sounds frightening’. Then they think about it and later that day or the following, they ask ‘when can we go home? You just have to plant a seed and they start to change focus.*”

*Physician, unit N*

### Staff ambivalence

#### Conflicting values and responsibilities

The respondents referred to parents’ desire to take their infant home as a motivating factor and emphasized that breastfeeding, parent–infant bonding and parental empowerment often seemed to improve when the family transitioned to home care.

“*Parents ask this as soon as they get here: ‘When can we go home?’ Homesickness, it is a strong emotion.*”

*RN, unit G*

They reflected on the effect that the hospital environment might have on parents’ psychological well-being, and had diverging ideas on how to best overcome this. Parents were in some instances advised to leave the hospital to spend time at home, while other arranged for families to stay in or near the unit. Attitudes and practices towards siblings in the unit also varied, from encouraging them to stay whenever they wanted to discouraging them and expressing concern that siblings would disturb the infant.

Both physicians and RNs expressed ambivalence about the optimal timing of the transition from hospital to home, balancing the risk of not being able to provide hands-on support should an adverse event occur. Physicians stated that most colleagues relied only on their own assessment, and that length of stay thus could vary by 1–2 weeks. The respondents also expressed ambivalence regarding the parents’ role in the infant’s care and views differed between and within units on whether parents should assume medical care and staff responsibilities. This affected what tasks and technology parents were entrusted with in the unit and at home.

#### What care can be moved to home?

When discharging infants at postmenstrual age (PMA) < 35 weeks, 8/36 (22%) units routinely used pulse oximetry or apnea alarm (Table 1) for home monitoring. Respondents from these units reported positive experiences and feedback from parents, were more open to delaying caffeine cessation until after discharge, and had established routines for doing so. In contrast, staff in units that did not use apnea monitoring at home expressed concerns that parents would both be hesitant to remove the device and suffer from alarm fatigue. Some questioned the safety of discontinuing caffeine treatment at home.

Providing breastfeeding support at home was mostly described as being easier than in the unit since mothers were perceived to be more relaxed and empowered at home. Information provided in NH was also described as more consistent than that provided in the unit.

“*In the unit, we have a major problem with parents receiving endless variations of breastfeeding information and strategies. In a single day, they may receive ten different suggestions, all with good intentions. It is usually the case that things become calmer and more consistent when they enter home care.”*

*Physician, unit N*

### Infant assessment

#### Evaluation of physiological stability

In general, evaluation of the infant’s physiological stability was found to be subjective, and there were difficulties in translating stability assessment criteria into clinical practice.

“*It is somewhat in the eye of the beholder. We don’t have any formal criteria for determining whether the infant is stable; it’s a matter of judgment. If I would write them [the criteria] down, they would be very vague. You wouldn’t have much use for it in clinical practice, because the assessment would be so individual anyway.*”

*Physician, unit H*

Cardiorespiratory stability and maintenance of normal body temperature without a heated mattress were minimum discharge requirements in all units (Table 1). The definition of cardiorespiratory stability varied, was described by respondents as vague, and additional criteria (Table 1) were used. Defined minimum PMA constituted a ‘safety barrier’ in 31/36 (86%) units, and one in five units used weight-based discharge criteria (Table 1). All respondents described an ongoing trend towards discharge at lower weight and PMA, without concomitant increase in number of re-admissions or adverse events.

Different structured pre-discharge tests (Table 1) were in use, perceived to aid decision-making but were not considered based on evidence. Accordingly, the assessment of infant stability was primarily based on the individual clinicians’ bedside observations, with input from bedside staff, and parents were not formally involved.

#### Feeding and nutrition

Practices varied between units as to whether the infant should be able to breast- or bottle feed a certain amount before transition to home care, or only considered to be respiratory stable during feeding (Table 1). While parents routinely handled tube feeds, which was perceived as empowering and important, nutritional planning was managed with little parental involvement. Timing of parental involvement in tube feeding varied depending on organizational routines and individual attitudes.

### Evaluation of parents’ competence in parenting

#### Vague criteria and guiding checklists

Although the decision to discharge was based primarily on the infant’s stability and readiness, 16/36 (44%) units also had requirements for parents (Table 1), yet discharge planning tools provided little guidance on how to assess parental readiness. Respondents emphasized parent’s presence and involvement in infant care, as means to achieve independence and confidence. Both facilities and staff attitudes could limit parents’ ability to stay overnight, but after transfer to a family room, most expected parents to stay with and care for their infant around the clock. Staff reflected on the parents’ ability to recognize their role and importance, and described how even experienced parents could need support to perform daily care and breastfeed.

“*You have to work very actively with some parents, to help them stay present. They don’t see their own importance in the infant’s development, but think that we fulfil that role. There are probably many parents who feel that they are somehow not needed, especially when there is a lot of intensive care.*”

*RN, unit Z*

#### Balancing infant and parent readiness

While mainly physicians were considered responsible for assessing infant readiness, RNs were primarily responsible for assessing parental readiness. Both professions reported that physicians often considered the family ready for discharge earlier than RNs. The ideal discharge preparation was described as a stepwise process, giving parents time to prepare, become motivated and assume full responsibility for all care and care planning. In daily practice, staff reported giving parents brief information at admission and physicians reported initiating the discharge process when they could predict an approximate discharge date. As a result, discharge-related information was often deferred until the infant met discharge criteria, leaving little time for parents to adjust. Respondents also described their experience that late or rushed information could cause parents to doubt the stability of their infant and the reliability of the staff’s assessment.

“*We can become better at initiating conversations about discharge, we say that all the time, but it’s challenging to predict the day. My experience is that parents are often faced with ‘well, today you can go home’ or they’re given very, very short notice. This has been the same in every unit I have worked in.*”

*Physician, unit Z*

“*We would like that even if we do not have a fixed date, we say ‘now you have a month to get ready and feel confident about going home’. That preparation is done step-by-step so that the parents feel ‘now we are really ready, and you [the physician] just have to say the word go and we will run’.*”

*RN, unit I*

#### Social aspects

The assessment of the family’s social situation was based primarily on staff’s perception of parents in the unit. Parents with social or psychological vulnerabilities were excluded from NH, as staff felt limited in their ability to assess and support them. Several respondents called for training and assessment tools in this area, both in the unit and at home.

Language barriers were considered significant in terms of getting information across and assessing parental readiness. In general, interpreters were rarely utilized during medical rounds or in the infant’s daily care. Although interpretation was perceived as easily accessible both in the unit and at home, 16/36 (44%) units excluded families who did not speak English or Swedish from NH (Table 1).

“*The assessment of whether the parents’ social situation is stable is also individual. It’s not that we check the home environment or so, it’s just the impression you get of the parents. On the other hand, some parents may not be at their best when they are hospitalized 24/7.*”

*RN, unit I*

### Staff-driven process for the family’s transition to home

#### Providing parental information

Respondents emphasized the importance of providing discharge information at an early stage of hospital admission, discussing it repeatedly with families, and adjusting information as the infant’s status changes. Physicians had limited knowledge about information given by RNs, and vice versa. This was reflected on as less optimal but acceptable, since both professions thought they complemented each other. All stressed the importance of enabling parents to see the healthy aspects of their infant. One unit described how group sessions in which NH staff provided information to families expected to be discharged within a couple of weeks, had facilitated for parents to support each other in overcoming concerns related to the transition. In addition, these sessions had improved timing of discharge preparation and reduced last-minute changes.

“*It has proved to be very important for home care to give information at an early stage. Why they are being admitted and what is expected in the near future, and then gradually try to build on that information while medical assessments and treatments are ongoing.*”

*RN, unit Y*

#### Skills training

The transition from intensive care to a family room was described as an important first step in transferring care responsibilities to parents and facilitating participation in daily care and handling of feeding tube and oral medications (Table 2). Formal training in how to manage adverse events (e.g. apnea), assess the infant’s clinical condition, and recognize signs of deterioration (Table 2) was in most units deferred until the infant reached a certain PMA or met discharge criteria.

The respondents reflected on the home-like situation when interacting with parents in a single-family room, requiring staff to be more confident in their professional role. They described how junior colleagues could be reluctant to enter the family room, not wanting to ‘disturb’ the family. This could delay breastfeeding support and skills training, and consequently, discharge planning. Home visits could be similarly demanding, although NH staff were in general more experienced and emphasized the benefits of interacting with and supporting the family at home.

“*In the intensive care unit, the parents visit their child, but as soon as they enter a single-family room, we’re visiting a family.*”

*Physician, unit N*

#### Flexible but inequal discharge planning

Several infant-related discharge criteria were flexible in selected situations. Exceptions from weight or feeding criteria (if applicable) were made if parents had older children at home, or during periods of restricted visitation. Proactive parents who asked for earlier discharge could be discharged with a scale or pulse oximeter, or instructed how to replace the feeding tube, even if this was not standard practice.

### Support at home

#### Adjust to being at home

A first contact or visit was commonly scheduled within 1–2 days after discharge, to support parents in the transition, and many units provided continuity through RNs or assistant nurses working both in NH and in the neonatal unit. Staff expressed NH in terms of ‘achieving normalization’, aiming for parents to overcome hospitalization and the perception of having a sick or vulnerable infant. Moving from fixed-interval to cue-based feeding and training parents to recognize common infant expressions was being an important part of this.

“*Home care can reduce the number of procedures and the hospitalization that parents experience when they are in the unit. We also talk a lot about healthy normal behavior. Many parents think that their infant is sick or has stomach pains when they start screaming at night, when they really just want to eat.*”

*RN, unit V*

#### Supportive practices at home

After the first week at home, most units individualized care and scheduled contacts or visits depending on the family’s needs. NH was provided through outpatient visits, home visits, or telemedicine (Table 3), often in combination. A parent-driven shift from home visits to telemedicine, with increased participation of both parents, was reported but in general, staff preferred to meet with the family in person ‘to build a relationship’ before moving on to using telemedicine. Telemedicine was described as appropriate for breastfeeding support and for assessment of the infant’s well-being. Staff also described how digital tools had increased access to NH. Removal of the feeding tube often marked the end of routine NH where after the responsibility for follow-up was transferred to regular child health care services.

“*Now we have our homecare, which has expanded and takes care of so many things. A lot of what we used to do in the unit is now done at home.*”

*RN, unit U*

## Discussion

In this study of current discharge practices of preterm infants in Swedish neonatal units we found a range of barriers and facilitators in the transition from hospital to home care. Staff expressed ambivalence about the optimal timing of discharge in relation to infant stability, parents’ roles and responsibilities, and the appropriate level of complexity of home care. In the absence of national guidelines, assessment criteria and tools varied (Table 1), were of limited value and lacked evidence base, leaving the evaluation to the discretion of the individual staff member. Defined minimum PMA constituted a ‘safety barrier’, and only one in five units used weight-based discharge criteria (Table 1). All respondents described an ongoing trend towards discharge at lower weight and PMA, without increased number of re-admissions and adverse events.

Staff were at times reluctant to involve parents in ‘professional’ care and decision-making, but also recognized that parents could struggle to find their role and emphasized parental presence in the unit. Daily care tasks were delegated but there was limited involvement in care planning and information was censored to protect parents from stress and disappointment, a phenomenon also reported by parents in a study by Franck et al.^21^ Several units postponed discharge information and skills training until the infant met discharge criteria. The transition process was staff driven and staff discontinuity and lack of training in how to interact with parents was perceived to cause delay. After discharge, NH aimed to normalize and support parents in taking responsibility for all aspects of infant assessment and care. Home care models combining telemedicine and home visits, with modes and intervals adapted to the needs and preference of the family, had been developed and was described as effective and appraised by both parents and staff.

Criteria that are not evidence-based may constitute unnecessary barriers to discharge and delay transition to home care without improving safety.^17^ As suggested by Schuler et al.^22^ and Moen,^23^ this might instead reduce the quality of care due to unnecessarily long hospital stays, with negative effects on breastfeeding and growth. Schuler et al.^22^ and Charpak et al.^24,25^ argue that circulatory, respiratory and thermoregulatory stability criteria may be sufficient, in combination with close follow-up and well-prepared parents. Lundberg et al.^26^ report a readmission rate of 5,2% during NH with no significantly increased risk associated with SGA, PMA, or weight at NH start.

Key components in pre-discharge preparation programs^27^ and guidelines include parental involvement in infant care,^11,28–32^ early and continuous information,^28,32–36^ mutual and visualized discharge planning,^11,34,37–39^ and special attendance to the needs of socially vulnerable families.^28,30,33,35,40^ Our findings show a lack of mutual and stepwise discharge planning, with the transition to home process initiated late, leaving little time for parents to adapt. This contrasts with a study by Ingram et al.,^34^ where parents describe visualization of the transition-to-home process and having a ‘working date’ for discharge as particularly important. In the unit, where the focus is on safety and competence in areas where staff are skilled, parental competencies risk being less valued^7^ and parental views and needs less prioritised.^41–43^ To overcome this, parents should be recognized as primary caregivers^11,44,45^ and their contribution facilitated during medical rounds.^11,38,42^ In a study by Franck et al.,^21^ most parents voiced that they should be actively involved in decision-making to build mutual trust and power sharing. Another study by Lorie et al.^46^ suggests that sharing information and decision-making becomes increasingly important as the time for discharge approaches. Creating a structure such as regular parental group sessions could help to improve the timing of discharge preparation, alter the dynamic between parents and staff, and enable peer support.^47^

Parents’ social situation, and language skills delayed discharge and limited access to NH for families who in other studies have been shown to most benefit from care in a home environment.^33,39^ This needs to be improved through increased use of interpreters and other tools for translation.^48^ Staff training in how to communicate with and support vulnerable families,^40^ and use of validated assessment tools,^49^ might facilitate both assessment and parents’ readiness for discharge per se.^40^

In the development of telemedicine services for neonatal homecare, Garne Holm et al.^15,50,51^ have shown that participatory methods can improve the design and help identify challenges associated with delivery of the service, based on the needs of parents and clinical staff. A pilot study by Bardach et al.^52^ indicates that using participatory methods to design pre-and post-discharge protocols, might also reduce the length of stay and promote patient- and parent-centered care.^52^

### Strengths and limitations

With a response rate of 100%, this study provides a comprehensive description of discharge practices and preparations in Swedish neonatal units. Having respondents outnumber the interviewer created a dynamic setting in which respondents reflected on each other’s answers, thereby enabling the interviewer to gain a clearer and deeper understanding. With joint interviews, we intended that the respondents would hold each other accountable and state their true praxis, but interviewing staff working closely together could also have added to social desirability bias. It should also be noted that family centered and integrated care can be considered standard in most Swedish units, and parental presence is facilitated by government financial support. This may not be the case in other countries and the implementation of early and continuous parental involvement as presented in this study may therefore be more challenging.

## Conclusion

Staff ambivalence about the optimal timing of discharge, stemming from varying individual experience and personal opinion, together with the lack of evidence, may contribute to the observed differences in discharge-related decision making. Establishing evidence-based protocols for the transition to home might reduce variations in practice. In addition, earlier involvement of parents in all aspects of pre-discharge preparation and care planning, including assessment of infant stability and parental readiness, may facilitate transfer of responsibility, build independence and reduce unnecessary discharge delay.

## Supplementary information

Supplementary information

## Data Availability

The datasets generated and analysed during the current study are available from the corresponding author on reasonable request.
