# Supplementary material for: Discharge criteria, practices, and decision-making in the transition of preterm infants to home
Source: Pediatr Res. 2024 Nov 27;99(2):670–7. doi: 10.1038/s41390-024-03752-w (PMC12956557; doi:10.1038/s41390-024-03752-w)
Supplement: Supplementary file 1 — Supplementary information [file 41390_2024_3752_MOESM1_ESM.pdf]

Interview guide TO HOME Study II

Discharge criteria, practices, and decision-making in the transition of preterm infants to home care.

Information to participants

Our aim is to study practices and preparations related to discharge and gain further understanding of facilitators and barriers in the transition to home process. We also want to explore how the units have structured support for families after discharge, including home-based care and collaboration with CHC services.

Survey on discharge criteria, parental education and post-discharge support

In the beginning of this interview, we will present ourselves and then start with a few questions regarding you, your unit and the units' structure of home care support.

Personal data:

- Profession
- Role and responsibilities in the unit and/or in home care
- Experience as a clinician, including present clinical role

Data on your unit:

- Number of births/years
- Number of beds (including if parents can stay overnight)
- Written discharge criteria and guidelines
- Standardized parental pre-discharge education (including information, preparation and training of procedures)
- Structure for readmission to neonatal unit and phone support (by neonatal staff) after discharge to home
- Structure of home care support (including staffing, digital practices, home visits, outpatient re-visits to neonatal staff, provided equipment at home)

Semi-structured interview

Introduction

Can you describe the process of transitioning preterm infants to home? How and when does it start in your unit?

Major topics

Can you tell us what the requirements are for a child to be discharged from your neonatal unit?

- *Are there infant-related discharge criteria?*
- *Are there parent-related discharge criteria?*
- *Are there other kinds of criteria or considerations?*

Can you describe how you work with parental involvement and participation in infant care in your unit?

- *How do you support parents in managing their infant's care?*
- *How do you involve parents in care planning?*
- *How do you support parents in assessing the infant's condition and wellbeing?*
- *What opportunities to families have to stay with their infant in the unit?*

Can you tell us what and how training and information is given to parents before the infant is discharged to home?

- *What practical procedures do families need to be able to perform in the unit? How do you support them in performing them?*
- *What practical procedures do families need to be able to perform at home? How do you support them in performing them?*
- *How do you involve parents in discharge planning?*

Can you describe how you work to support infants and parents to breastfeed and how you plan the infant's nutrition?

- *How do you support infants and parents to breastfeed; in the unit and at home?*
- *How and when are the infant's feeding and nutrition planned; during the hospital stay and at home?*

Can you describe the structure of your neonatal home care and/or outpatient follow up?

- *How (during what days and hours) can parents have access neonatal home care?*
- *What professions are involved ?*
- *Do you have regular rounds and how are they performed?*
- *How do you communicate with families at home?*

- *Are there any restrictions to or exclusion criteria in home care?*
- *What kind of care can parents provide at home?*
- *How do you use alarms or other technical surveillance in the home?*
- *Is digital support available?*
- *If the child needs to be readmitted, how will you arrange this?*
- *When and how is the infant discharged from neonatal homecare?*

How do you co-operate with CHC services?

- *Is there cooperation during the infant's care in the unit and/or before discharge?*
- *When and how is the family's first contact with the CHC after discharge?*
- *Which issues are dealt with by your neonatal home care service and which are dealt with by CHC?*

Is there anything that prevents you from discharging infants earlier?

Is there anything that would encourage you to discharge infants earlier?

Is there anything you are particularly proud of that you would like to tell us about?

Is there anything else you would like to add?
